# Supplementary material for: Identification of Inflammatory Proteomics Networks of Toll-like Receptor 4 through Immunoprecipitation-Based Chemical Cross-Linking Proteomics
Source: Proteomes. 2022 Sep 1;10(3):31. doi: 10.3390/proteomes10030031 (PMC9506174; doi:10.3390/proteomes10030031)
Supplement: Supplementary file 1 [file proteomes-10-00031-s001.zip › Supplementary Materials.pdf]

Identification of Inflammatory Proteomics Networks of Toll-like Receptor 4 through Immunoprecipitation-Based Chemical Cross-Linking Proteomics

A.D.A. Shahinuzzaman [1,2], Abu Hena Mostafa Kamal [1,3], Jayanta K. Chakrabarty [1,4], Aurchie Rahman [1] and Saiful M. Chowdhury [1,*]

[1] *Department of Chemistry and Biochemistry, The University of Texas at Arlington, Arlington, TX 76019, USA; ada.shahinuzzaman@mavs.uta.edu (A.S.); abuhenamostafa.kamal@bcm.edu (A.H.M.K.); jayanta.chakrabarty@mavs.uta.edu (J.K.C.); aurchie.rahman@mavs.uta.edu (A.R.)*

[2] Pharmaceutical Sciences Research Division, Bangladesh Council of Scientific and Industrial Research (BCSIR), Dhaka 1205, Bangladesh

[3] Advanced Technology Cores, Dan L Duncan Comprehensive Cancer Center, Baylor College of Medicine, Houston, TX 77030, USA

[4] Quantitative Proteomics and Metabolomics Center, Columbia University, New York, NY 10027, USA

* Correspondence: schowd@uta.edu; Tel.: +1-817-272-5439

Supplementary Figures

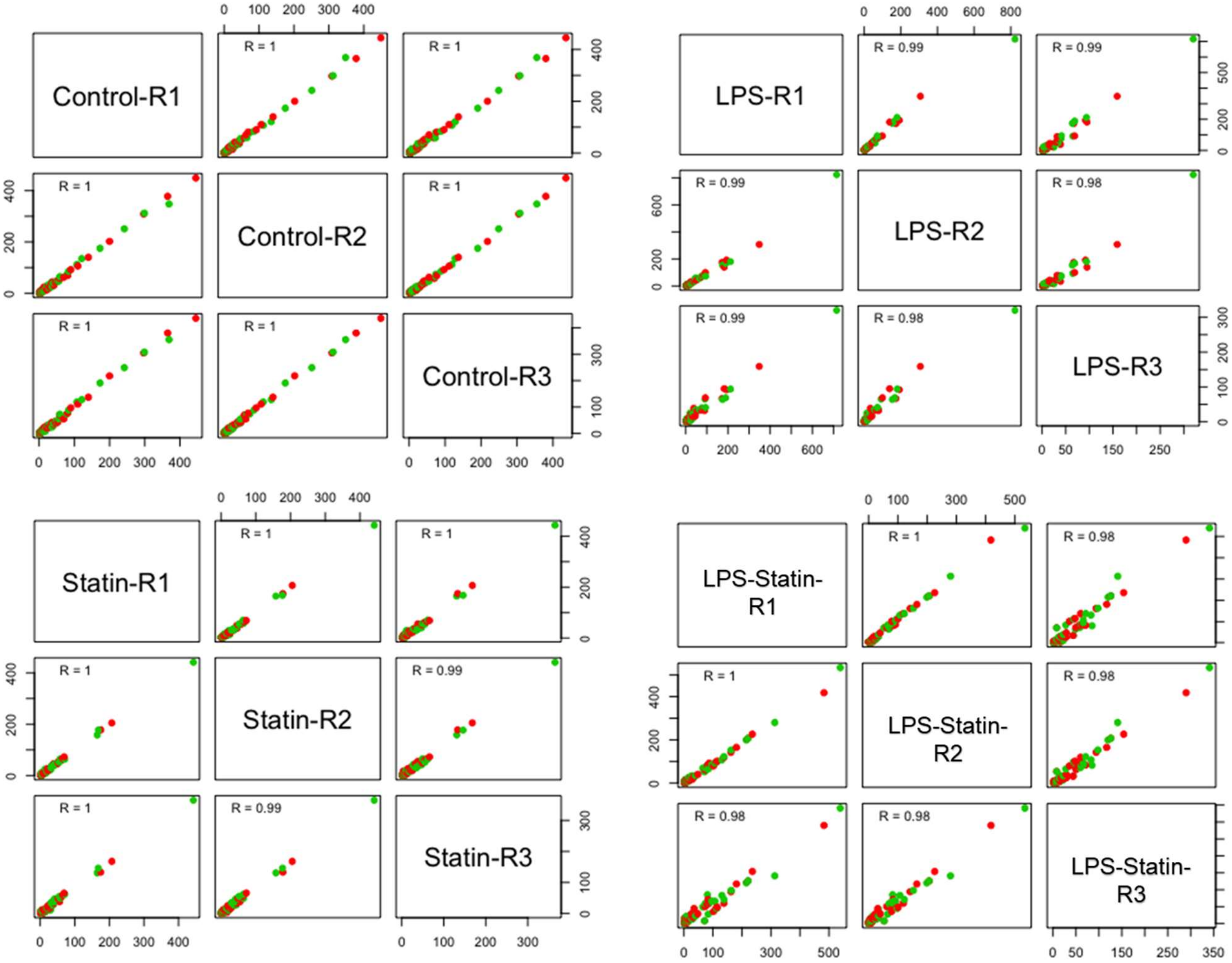

Figure S1. Scatter plots and pairwise correlations among the biological replicates showing significant correlation patterns among replicates of Control sample, LPS, Statin and LPS-Statin treated samples without cross-linker. The PSMs (Peptide Spectral counts) of replicates are plotted against each other across x and y-axis, correspondingly. Every spot symbolizes the abundances of a proteins and corresponds to Pearson's correlation coefficient (R^2) of 1. The scatter plot and pairwise correlation analysis was performed by R package ver. 3.5.3.

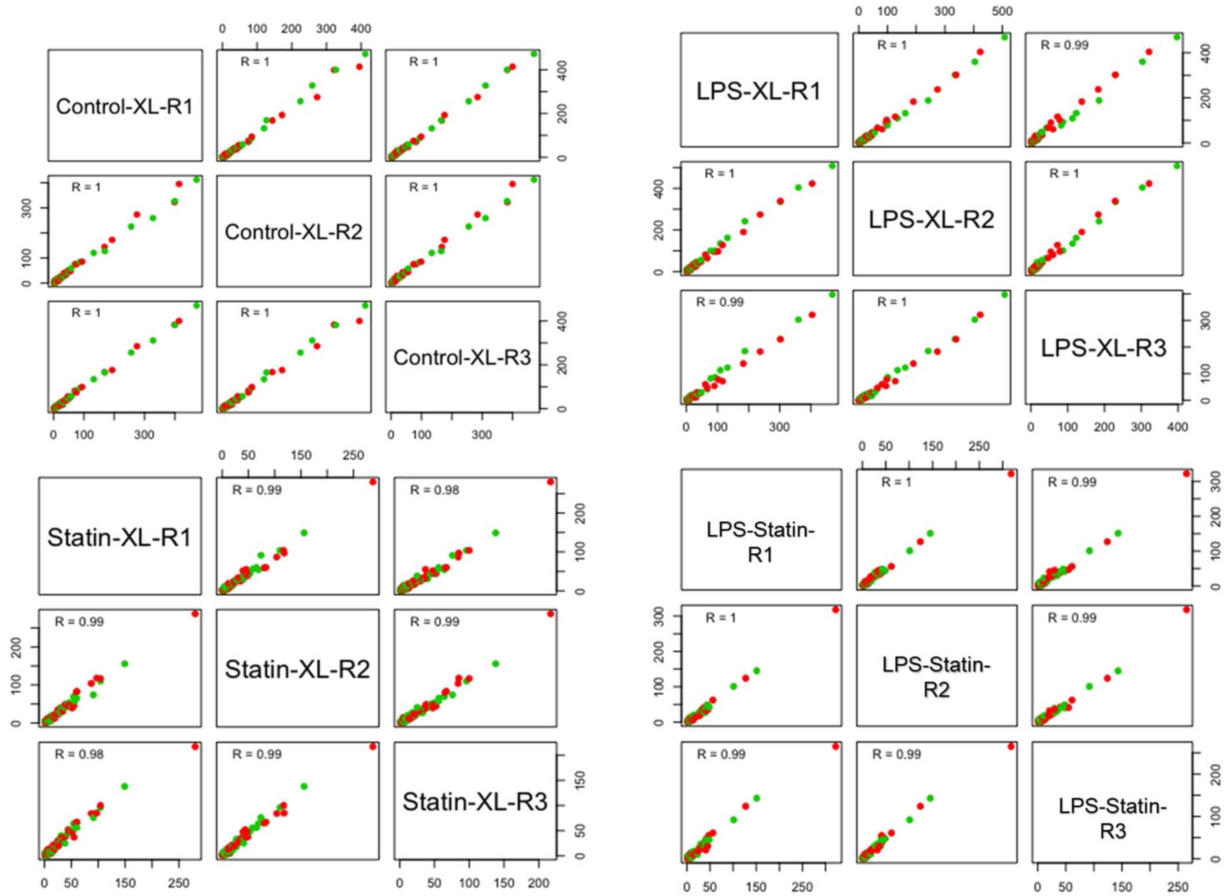

Figure S2. Scatter plots and pairwise correlations among the biological replicates showing significant correlation patterns among replicates of Control sample, LPS, Statin and LPS-Statin treated samples with cross-linker treatment. The PSMs (Peptide Spectral counts) of replicates are plotted against each other across x and y-axis, correspondingly. Every spot symbolizes the abundances of a proteins and corresponds to Pearson's correlation coefficient (R^2) of 1. The scatter plot and pairwise correlation analysis was performed by R package ver. 3.5.3.

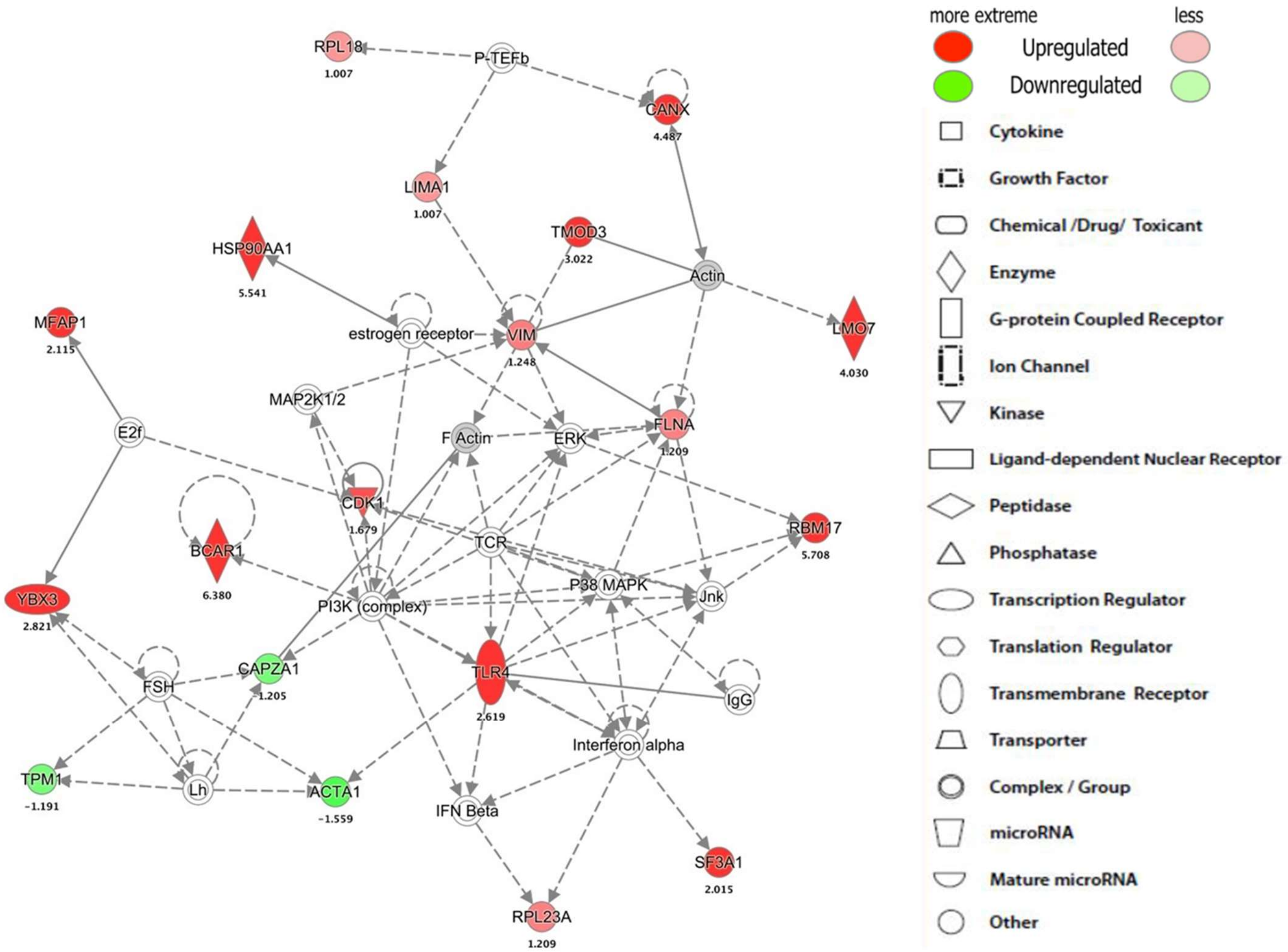

Figure S3. TLR4 targeted protein network with expression profile. The interaction network shown was generated using IPA bioinformatics software upon treatment of LPS in HA-TLR4-HEK293 cells.

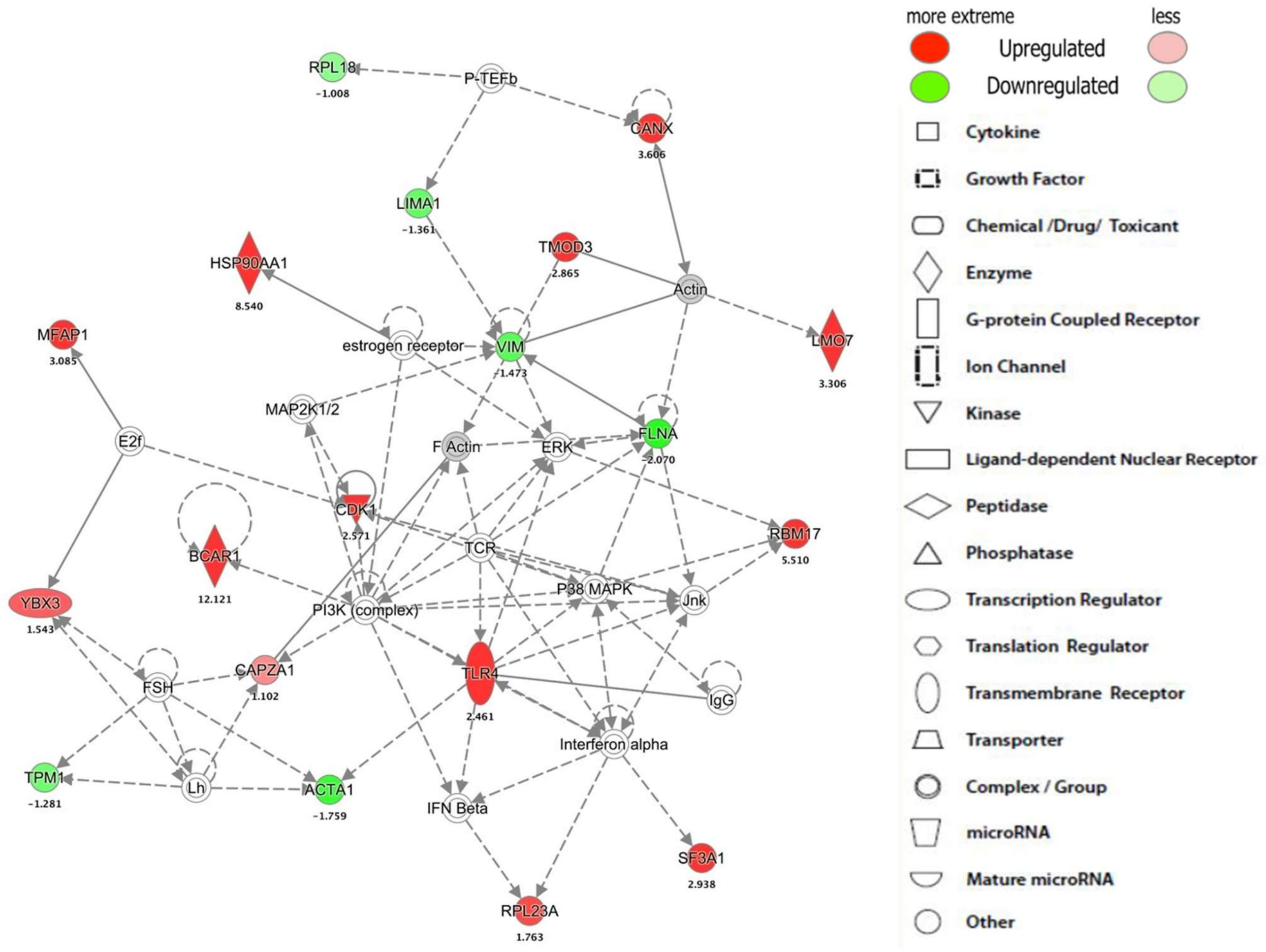

Figure S4. TLR4 targeted protein network with expression profile. The interaction network shown was generated using IPA bioinformatics software upon treatment of LPS-Statin in HA-TLR4-HEK293 cells.

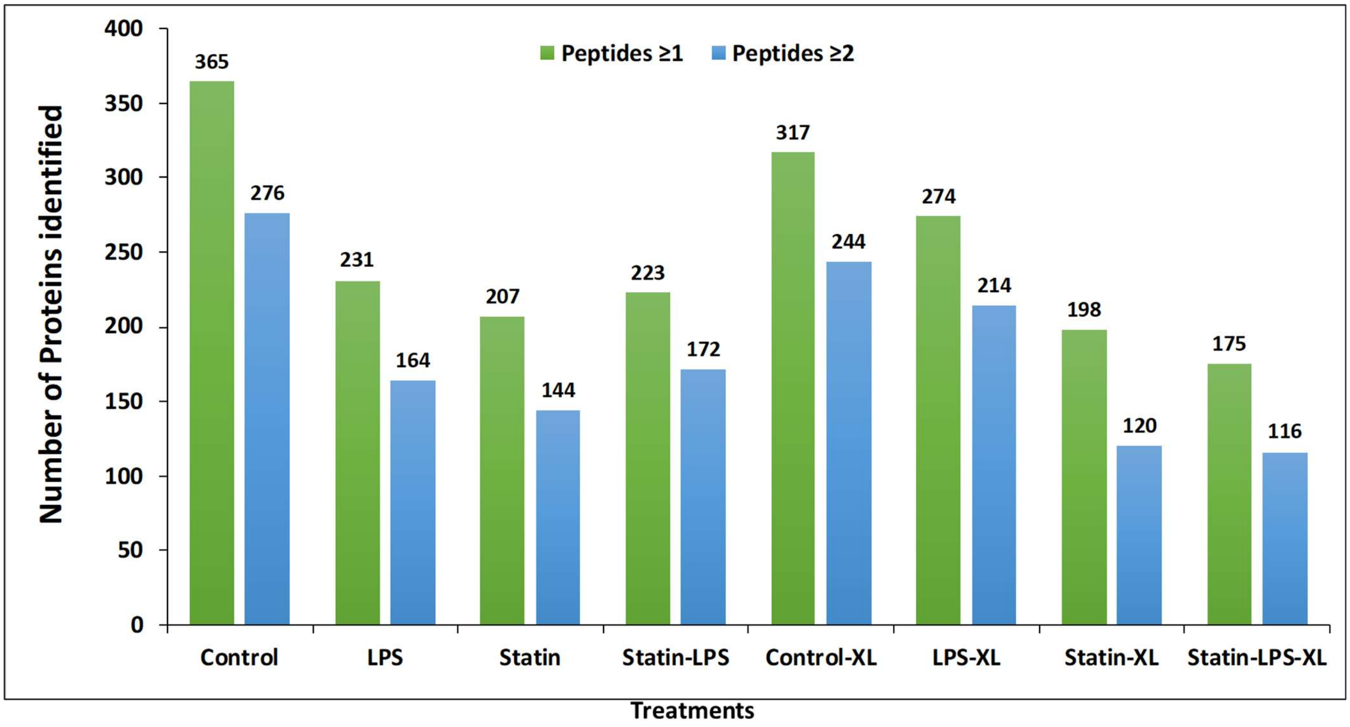

Figure S5. The number of proteins identified with or without cross-linkers across each treatment conditions where at least 1 or 2 peptides identified for each sample.
